# Bi-Allelic Mutations in STXBP2 Reveal a Complementary Role for STXBP1 in Cytotoxic Lymphocyte Killing

**DOI:** 10.3389/fimmu.2018.00529

**Published:** 2018-03-15

**Authors:** Jamie A. Lopez, Tahereh Noori, Adrian Minson, Lu Li Jovanoska, Kevin Thia, Michael S. Hildebrand, Hedieh Akhlaghi, Phillip K. Darcy, Michael H. Kershaw, Natasha J. Brown, Andrew Grigg, Joseph A. Trapani, Ilia Voskoboinik

**Affiliations:** ^1^Cancer Immunology Program, Peter MacCallum Cancer Centre, Melbourne, VIC, Australia; ^2^Sir Peter MacCallum Department of Oncology, The University of Melbourne, Parkville, VIC, Australia; ^3^Department of Clinical Haematology, Austin Health, Heidelberg, VIC, Australia; ^4^Department of Medicine, Austin Health, Heidelberg, VIC, Australia; ^5^Department of Clinical Genetics, Austin Health, Heidelberg, VIC, Australia

**Keywords:** familial haemophagocytic lymphohistiocytosis, cytotoxic lymphocytes, immunodeficiency, apoptosis, natural killer cells, cytotoxic T cells, Munc18-2, Munc18-1

## Abstract

The ability of cytotoxic lymphocytes (CL) to eliminate virus-infected or cancerous target cells through the granule exocytosis death pathway is critical to immune homeostasis. Congenital loss of CL function due to bi-allelic mutations in *PRF1, UNC13D, STX11*, or *STXBP2* leads to a potentially fatal immune dysregulation, familial haemophagocytic lymphohistiocytosis (FHL). This occurs due to the failure of CLs to release functional pore-forming protein perforin and, therefore, inability to kill the target cell. Bi-allelic mutations in partner proteins *STXBP2* or *STX11* impair CL cytotoxicity due to failed docking/fusion of cytotoxic secretory granules with the plasma membrane. One unique feature of STXBP2- and STX11-deficient patient CLs is that their short-term *in vitro* treatment with a low concentration of IL-2 partially or completely restores natural killer (NK) cell degranulation and cytotoxicity, suggesting the existence of a secondary, yet unknown, pathway for secretory granule exocytosis. In the current report, we studied NK and T-cell function in an individual with late presentation of FHL due to hypomorphic bi-allelic mutations in *STXBP2*. Intriguingly, in addition to the expected alterations in the STXBP2 and STX11 proteins, we also observed a concomitant significant reduction in the expression of homologous STXBP1 protein and its partner STX1, which had never been implicated in CL function. Further analysis of human NK and T cells demonstrated a functional role for the STXBP1/STX1 axis in NK and CD8+ T-cell cytotoxicity, where it appears to be responsible for as much as 50% of their cytotoxic activity. This discovery suggests a unique and previously unappreciated interplay between STXBP/Munc proteins regulating the same essential granule exocytosis pathway.

## Introduction

Natural killer (NK) cells and CD8+ T cells, collectively known as cytotoxic lymphocytes (CLs), serve a vital role in immune surveillance by identifying virus-infected and malignant cells and eliminating them through perforin and granzyme-mediated apoptosis (1). The degranulation of CL secretory granules is a critical physiological process to enable perforin and granzyme release into the immune synapse. Bi-allelic mutations in Munc13-4, or the partner proteins Syntaxin-11 or STXBP2, result in defective degranulation and cytotoxicity of NK cells, leading to Familial Haemophagocytic Lymphohisitiocytosis (FHL) (2).

Syntaxin-11 and STXBP2 biochemically interact within the cell (3, 4) and are, therefore, likely to function at the same step in secretory granule exocytosis—the docking and fusion of the granules with the plasma membrane. Recent work by Spessott and colleagues confirmed that STXBP2 directly stimulates the Syntaxin-11-mediated fusion of membranes (5).

STXBP2-deficient patient NK cells have severely reduced/absent degranulation but, surprisingly, and for reasons that are not clearly understood, short-term *in vitro* treatment of NK cells from STXBP2 (or Syntaxin-11) deficient patients with a low concentration of IL-2 partially or completely restores NK cell degranulation and cytotoxicity (3, 4, 6), suggesting the existence of a secondary pathway for secretory granule docking. Consistent with these observations, STXBP2/Syntaxin-11-deficient patients develop FHL slightly later than those who harbor mutations in non-redundant proteins, perforin, or Munc13-4 (7, 8).

In the current report, we studied NK and T-cell function in an individual with late presentation of FHL due to bi-allelic mutations in *STXBP2*. Intriguingly, in addition to the expected alterations in STXBP2, we also observed a concomitant reduction in the STXBP1 protein, encoded by the paralog, *STXBP1*. Further analysis of human NK and T cells demonstrated a functional role for STXBP1 in NK and cytotoxic T-cell cytotoxicity and suggests a unique interplay between STXBP/Munc18 proteins regulating the same exocytic event.

## Case Report

A 45-year-old female was admitted to hospital with mild pancytopenia and hepatosplenomegaly. Subsequently, CD3+ T-cell infiltrates were found in the bone marrow, with demonstration of a clonal population by T-cell receptor gene rearrangement analysis suggesting T-cell lymphoma. CHOP (two cycles) and then CHEOP (four cycles) therapy were given with a partial response and an autologous stem cell transplantation was planned 2 weeks after stem cell mobilization with G-CSF (5 µg/kg for 10 days). She developed with rapidly progressive pancytopenia with Hb 90 g/L (reference range 115–165), platelets 8 × 10^9^/L (reference range 150–400) and neutrophils 0.4 × 10^9^/L (reference range 2–7.5), ferritin >16,000 μg/L (reference range 30–150), and sCD25 >60,000 U/mL (reference range <2,400). In this context, FHL was suspected; on further review, it was noted that her brother died in his mid 30s from an illness with some features of FHL. The patient also suffers from focal epilepsy. Subsequent Sanger sequencing of *STXBP2* identified two mutations (encoding STXBP2 protein): c.1001C > T (p.P334L) and c.474_483del_insGA (p.C158Wfs*78). While the segregation analysis was not possible, given an apparent familial history of the disease (and the functional results shown below), it is highly likely that the patient had bi-allelic *STXBP2* mutations. No mutations were identified in *UNC13D, STX11*, or *PRF1*. Monthly Alemtuzumab (Campath) therapy for 6 months partially controlled her symptoms. At the time of writing this report, the patient was 4 months after a T-cell-depleted unrelated donor allograft with no evidence of HLH, but with the complication of multi-drug resistant CMV disease.

## Materials and Methods

### Reagents

Antibodies used were obtained as follows: rabbit polyclonal STXBP1, rabbit polyclonal STXBP2, rabbit monoclonal STXBP3 (Abcam, Cambridge, UK); rabbit polyclonal Syntaxin-4, rabbit polyclonal Syntaxin-11 (Proteintech, Rosemont, IL); mouse monoclonal Syntaxin-1, rabbit polyclonal Syntaxin-3 (Synaptic Systems, Gottingen, Germany); mouse monoclonal β-actin (Sigma-Aldrich, St. Louis, MO, USA); mouse monoclonal myc-tag (9B11) (Cell Signaling Technology, Danvers, MA, USA); goat anti-mouse F(ab’)2 IgG PE-conjugated (eBioscience); human CD3-FITC, human CD8 PerCP-cy 5.5, human CD16-Pacific BlueTM (BD Bioscience, San Jose, CA, USA); human CD3 (Orthoclone OKT3), human CD28 and human CD107a/Lamp1-Alexa488 (Biolegend, San Diego, CA, USA). All chemicals were from Sigma-Aldrich (St. Louis, MO, USA). The PG13 retroviral packaging cell line was from American Type Culture Collection (Manassas, VA, USA).

### Cell Culture

KHYG1 cells were maintained in RPMI (GIBCO, Life Technologies) supplemented with 10% (v/v) heat-inactivated FCS, 2 mM l-glutamine and 450 U/mL of human rIL-2. Human peripheral blood mononuclear cells (PBMCs) were maintained in RPMI supplemented with 10% (v/v) heat-inactivated FCS, 2 mM l-glutamine, 0.1 mM non-essential amino acids, 1 mM sodium pyruvate.

### T-Cell Activation

Peripheral blood mononuclear cells from a healthy donor control and the affected individual were activated with 30 ng/mL of anti-CD3 antibody and 600 U/mL IL-2 for 3 days and then cultured in the presence of 600 U/mL of human rIL-2.

### Munc18 Knockdown—KHYG1 Human NK Cells

Target sequence selection and shRNA-miR30 construction was performed as described previously (9, 10). Each miR30-based STXBP1, STXBP2, and STXBP3 shRNAs were subcloned into the pLMS-eBFP, pLMP-mCherry, and pLMS-GFP retroviral expression, respectively. The following 21-mer sequences were used to target human STXBP1 (#1 5′-GCTGCAAGATGACAGACATCA-3′; #2 5′-GCAAGATGACAGACATCATGA-3’), human STXBP2(#1 5′-GGACAAGGCGAACATCAAAGA-3′, #2 5′-GGCGAACATCAAAGACCTATC-3′, #3 5′-CCCTTTCCAGAGAAATAAACT-3’), human STXBP3 (5′-GATCCAGAATGTAAAGATAGA-3’). A scrambled target sequence (5′- TCTCGCTTGGGCGAGAGTAAG-3’) was used as the control shRNA and expressed in each of the matching expression vectors pLMS-eBFP, pLMP-mCherry, and pLMS-GFP. Amphotropic retrovirus was packaged in HEK293 cells, following calcium-phosphate transfection of the viral expression plasmid and amphotropic plasmid. The supernatant containing the virus was collected at 48 h post transfection and centrifuged at 2000 × *g* for 1 h onto RetroNectin-coated (Takara Bio-USA Inc., Mountain View, CA, USA) 24-well plates. Following a 2-h incubation at 37°C/5%CO_2_, 5 × 10^5^ KHYG1 cells were added to each well, centrifuged at 1000 × *g* for 1 h at RT and then incubated at 37°C/5%CO_2_. At 5–7 days post-transduction, flow cytometry was used to sort eBFP-, mCherry-, or GFP-positive cells expressing the shRNAs.

### CAR Transductions

Amphotropic virus encoding the chimeric antigen receptor anti-erbB2 CD28ζ was produced from the PG13 packaging cells (11) and used to transduce activated T cells following the viral transduction protocol described for KHYG1 cells. CAR-T positive cells were isolated by flow cytometry using an antibody against the surface exposed c-myc epitope and anti-mouse PE-conjugated.

### Munc18 Knockdown—Primary Human CAR-T Cells

Peripheral blood mononuclear cells were isolated from a healthy donor control, transduced with virus expressing either scrambled shRNA or STXBP1 shRNA and sorted based on the expression of the BFP reporter. BFP+ cells were subsequently transduced with virus expressing the CAR-T. CAR-T negative and CAR-T positive cells were sorted based on the expression of the surface expression of the myc epitope. CAR-T negative CD3^+^CD8^+^CD4^−^ cells were immuno-blotted for STXBP1 to determine knockdown. CAR-T positive cells were used to assess CAR-T killing function using ^51^Cr release assay, and the effector/target ratio was normalized for % CD3^+^CD8^+^CD4^−^ cells; CAR-T^+^CD4^+^ cells had marginal (<5%) cytotoxic activity compared to CAR-T^+^CD3^+^CD8^+^CD4^−^ cells.

### STXBP2-Knockout Primary Human T Cells

Peripheral blood mononuclear cells from a healthy donor control were activated and transduced with FUCas9Cherry and doxycycline inducible sgRNA encoded in pFH1tUTG GFP vector (#1 5′- tcccGCCCTCGGGGCTGAAGGCGG-3′, #2 5′- tcccTGAGCTAGGCCGCTCTCGTC-3′, #3 5′-tcccACCACCGCCTTCAGCCCCGA-3′) (12). Following guide expression, by incubating the cells with doxycycline for 7 days, GFP and Cherry double-positive and double-negative cells were isolated by flow cytometry.

### Degranulation Assays

CD107a/Lamp-1 externalization was used to determine NK and T-cell degranulation. Briefly, CAR-T cells were incubated in the presence or absence of MDA-MB-231 Her2 expressing target cells at 1:2 E:T ratio for 3 h at 37°C/5% CO_2_. NK cells were incubated with K562 targets at 1:2 E:T ratio for 3 h at 37°C/5% CO_2_ in the absence or presence of 100 U/mL of human IL-2. CD107a externalization was assessed in CD3−CD16+CD56+cells; spontaneous externalization of CD107a was assessed over 3 h in the absence of target cells.

### DNA Extraction, PCR, and Sanger Sequencing

Whole venous blood was obtained and genomic DNA extracted using a QIAamp DNA Maxi Kit (Qiagen, Valencia, CA, USA). Coding exons and splice sites of the STXBP1 gene (Chromosome 9:130, 374, 486–130, 454, 995; NM_003165; ENST00000373302.7) were sequenced. Regions were amplified using gene-specific primers designed to the reference human gene transcript (http://www.ncbi.nlm.nih.gov/gene). Primer sequences are available upon request. Amplification reactions were cycled using a standard protocol on a Veriti Thermal Cycler (Applied Biosystems, Carlsbad, CA, USA). Bidirectional sequencing of all exons and flanking regions, including splice sites was completed with a BigDyeTM v3.1 Terminator Cycle Sequencing Kit (Applied Biosystems), according to the manufacturer’s instructions. Sequencing products were resolved using a 3730xl DNA Analyzer (Applied Biosystems). All sequencing chromatograms were compared to published cDNA sequence; nucleotide changes were detected using Codon Code Aligner (CodonCode Corporation, Dedham, MA).

### Cytotoxicity Assays

Natural killer (NK) and CAR-T cell killing function was measured using standard chromium (^51^Cr) release assays, as described previously (13).

### Statistical Analyses

Statistical analyses (as shown in the Figure legends) were performed using GraphPad Prism 7 software.

### Ethics

Studies involving human cells were approved by the Peter MacCallum Cancer Centre Human Ethics Committee (approval number 12/73). The affected individual provided informed consent for publication.

## Results

**Figure 1:**
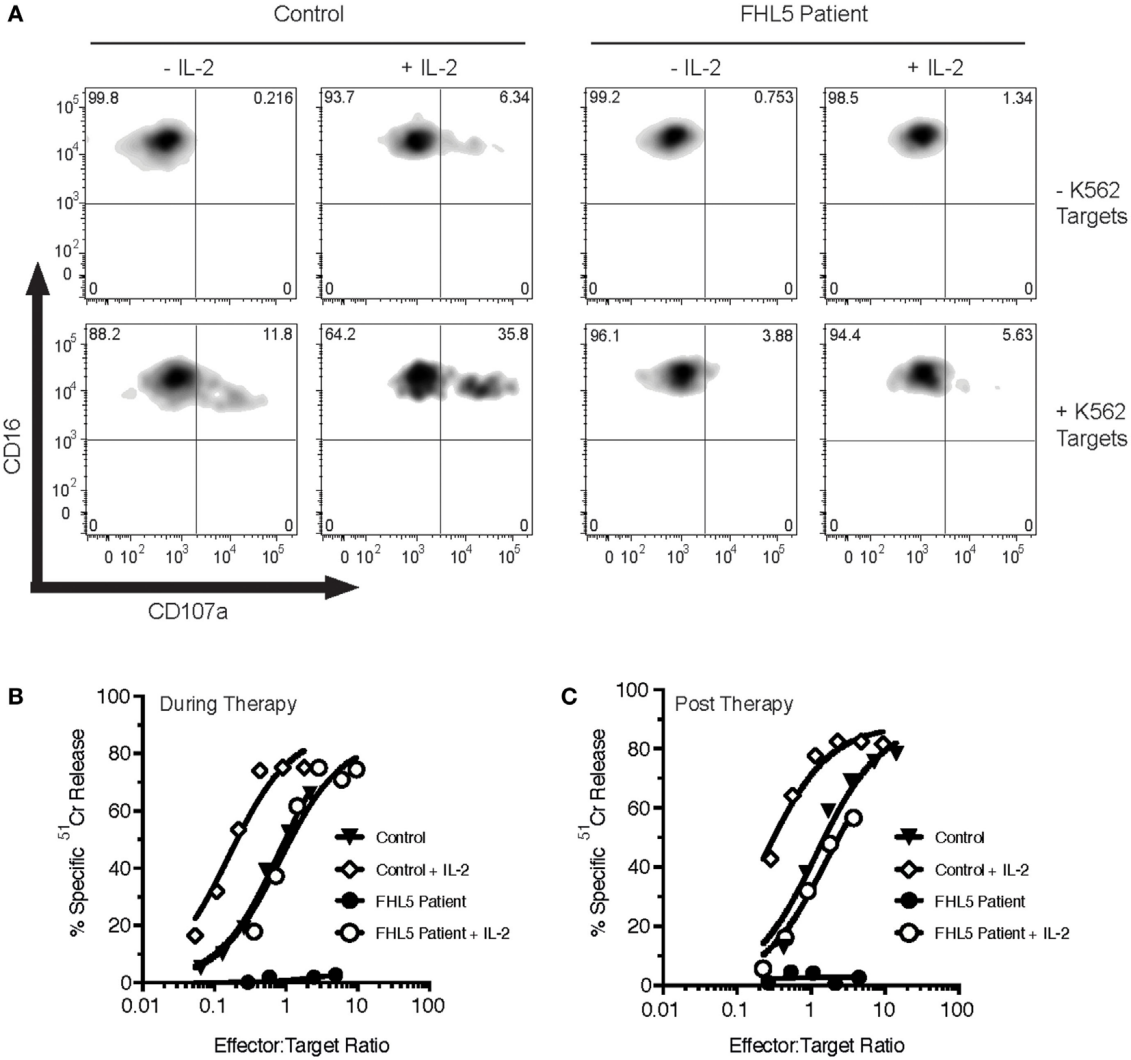
Bi-allelic mutations in *STXBP2* impair natural killer (NK) degranulation and cytotoxicity, which is partially restored by IL-2. Peripheral blood mononuclear cells (PBMCs) were isolated from a healthy donor control (Control) and an FHL5 patient and incubated for 16 h in the absence (-IL-2) or presence (+IL-2) of 100 U/mL of human IL-2. **(A)** PBMCs were incubated for 3 h in the absence (−K562) or presence (+ K652) of K562 target cells. NK degranulation was assessed by measurement of CD107a surface labeling in the CD16+ cell populations. Data are representative of two independent experiments. **(B,C)** PBMCs taken during therapy **(B)** or after therapy **(C)** were incubated with ^51^Cr-labeled K562 target cells for 4 h at the indicated effector to target cell ratios (normalized for the% of NK cells). NK cytotoxicity was determined by the release of ^51^Cr from target cells. Data are representative of two independent experiments.

**Figure 2:**
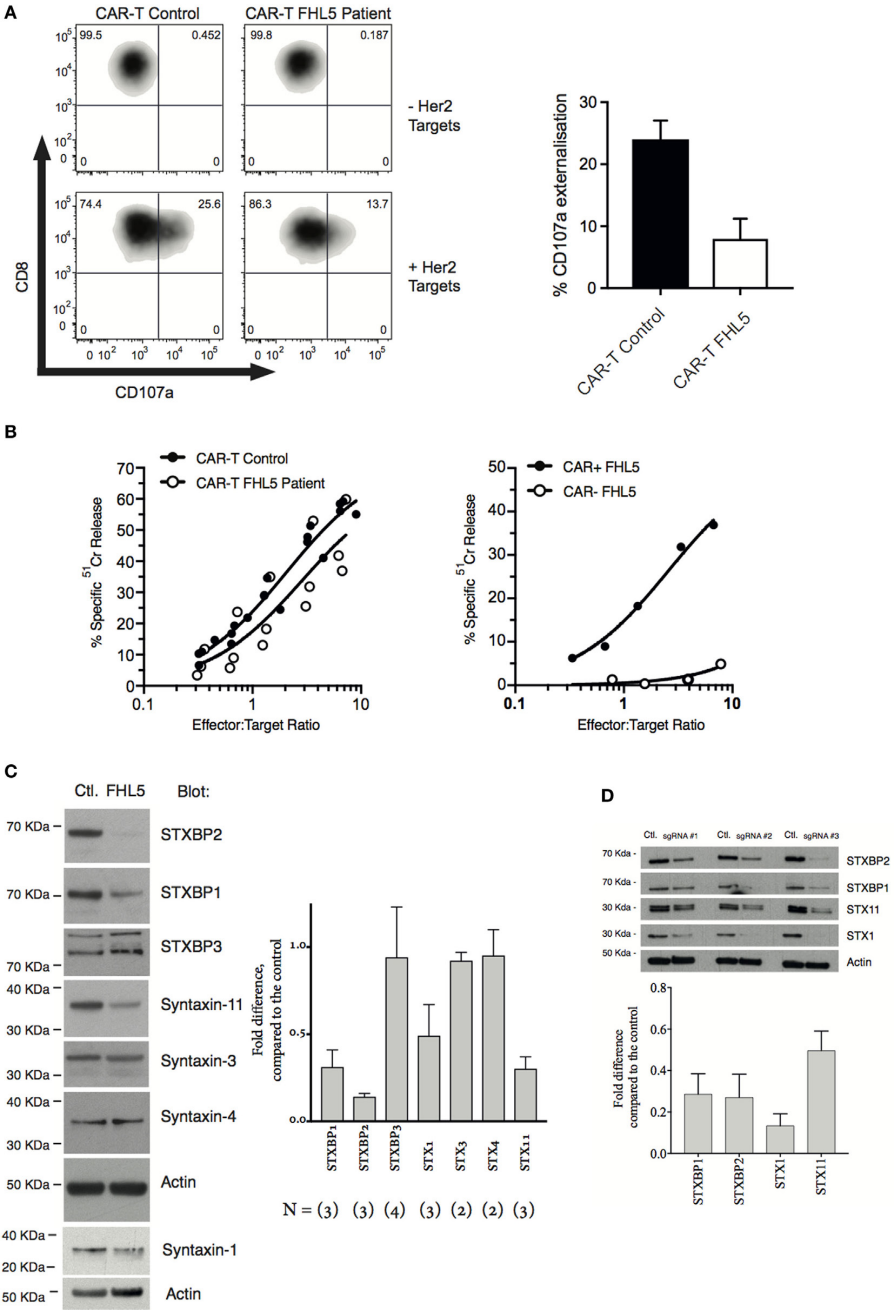
*STXBP2* mutations inhibit T-cell degranulation and perturb the expression of the STXBP1 isoform. **(A)** Peripheral blood mononuclear cells (PBMCs) were isolated from a healthy donor control (CAR-T Control) or an FHL5 patient (CAR-T FHL5 Patient) and incubated with 30 ng/mL of anti-CD3 antibody and 600 U/mL of IL-2 for 3 days. Cells were transduced with virus expressing a CAR specific for the Her2 antigen. CAR transduced PBMCs were incubated for 3 h in the absence (−Her2 Targets) or presence (+Her2 Targets) of MDA-MB-231 target cells overexpressing human Her2. CD8+CAR− T-cell degranulation was assessed by measurement of CD107a surface labeling in the CD8+ cell populations. The right panel shows the % of cells labeled for CD107a after incubation with Her2 targets. Data represent the mean ± SEM (*n* = 3 experiments). **(B)** CAR-T-expressing Control and FHL5 cells were incubated with ^51^Cr-labeled MDA-MB-231 Her2 expressing target cells for 4 h at the indicated effector to target cell ratios (normalized for the % of CD8+ T cells). CAR-T cytotoxicity was determined by the release of ^51^Cr from target cells. Data are representative of two independent experiments. The right panel shows the cytotoxicity of FHL5 CD8+ T cells in the absence (CAR−) and presence (CAR+) of CAR expression. Data are representative of two independent experiments. **(C)** PBMCs were isolated from a healthy donor control (Control) or an FHL5 patient and incubated with 30 ng/mL of anti-CD3 antibody and 600 U/mL of IL-2 for 3 days. CD3+CD8+ T cells were isolated by flow cytometry and whole cell lysates were blotted for STXBP1, STXBP2, STXBP3, STX1, STX3, STX4, STX11, and actin (loading control). Molecular weight standards (KDa) are indicated. The right panel shows the protein expression (% of Control) for each of the proteins in the FHL5 samples after each blot was normalized for actin expression. Data represent the mean ± SD (*n* = 2–3 experiments). **(D)** PBMCs were isolated from a healthy donor and incubated with 30 ng/mL of anti-CD3 antibody and 600 U/mL of IL-2 for 3 days. Cells were transduced with virus expressing Cas9 and the sgRNAs #1, #2, and #3. GFP^+^ Cherry^+^ and GFP^−^ Cherry^−^ control (Ctl) cells were isolated by flow cytometry and whole cell lysates were blotted for STXBP1, STXBP2, Syntaxin-11, Syntaxin-1, and actin (loading control). Molecular weight standards (KDa) are indicated. The panel below shows the protein expression (% of Control) for each of the proteins after each blot was normalized for actin expression. Data represent the average of three guides ± SEM (*n* = 3).

The patient’s NK cell function was assessed using the Lamp-1 externalization assay, a measure of degranulation, and ^51^Cr release assays that assesses NK cytotoxicity. Three measurements were made, twice prior to her monthly Alemtuzumab injection (“during therapy”) and once 8 weeks after the last Alemtuzumab injection (“post-therapy”). At each of these time points, the patient’s naïve NK cells showed marginal activity (Figures 1A–C). Overnight treatment with 100 U/mL IL-2 only partially restored NK cell degranulation and cytotoxicity. In order to determine the functional consequence of *STXBP2* mutations on primary human cytotoxic T-cell function, we employed a recombinant chimeric antigen T-cell receptor (CAR) technology. Primary CD3+CD8+ cells from the patient and healthy donors were transduced with an anti-HER2 CAR-T construct, and their activity was assessed against HER2+MDA-MB-231 target cells. In comparison to the controls, the patient cytotoxic T cells had reduced degranulation and cytotoxic activity (Figures 2A,B).

As expected, bi-allelic mutations in *STXBP2* resulted in a significant loss of STXBP2 protein relative to the control (Figure 2C). Consistent with previous reports, we also observed a decrease in the cognate t-SNARE Syntaxin-11, but not cognate Syntaxin-3. Interestingly, we found a significant decrease in the level of STXBP1 and its cognate t-SNARE Syntaxin-1. No differences in STXBP3 or syntaxin-4 protein levels were observed, suggesting a specific disruption to the STXBP2 and STXBP1 pathway/s (Figure 2C). Sequencing of the 19 coding exons and splice sites of the *STXBP1* gene in our patient did not reveal any pathogenic mutations nor were any single nucleotide polymorphisms detected. To replicate these findings in a recombinant setting, we knocked out *STXBP2* in primary human CD8+ T cells using CRISPR/cas9, using three guide RNAs. The results confirmed our original observation, demonstrating significant protein reductions in STX11, STXBP1, and STX1 protein levels as a consequence of STXBP2 loss (Figure 2D). A previous STXBP2/Syntaxin-11 structural study by Hackman et al. (14) demonstrated that, similar to STXBP2, STXBP1 was also capable of binding to Syntaxin-11, albeit at a lower affinity. They speculated that STXBP1 could compensate in the absence of functional STXBP2. Our data suggests that this may not be the case since STXBP1 protein expression appears to be dependant on STXBP2.

**Figure 3:**
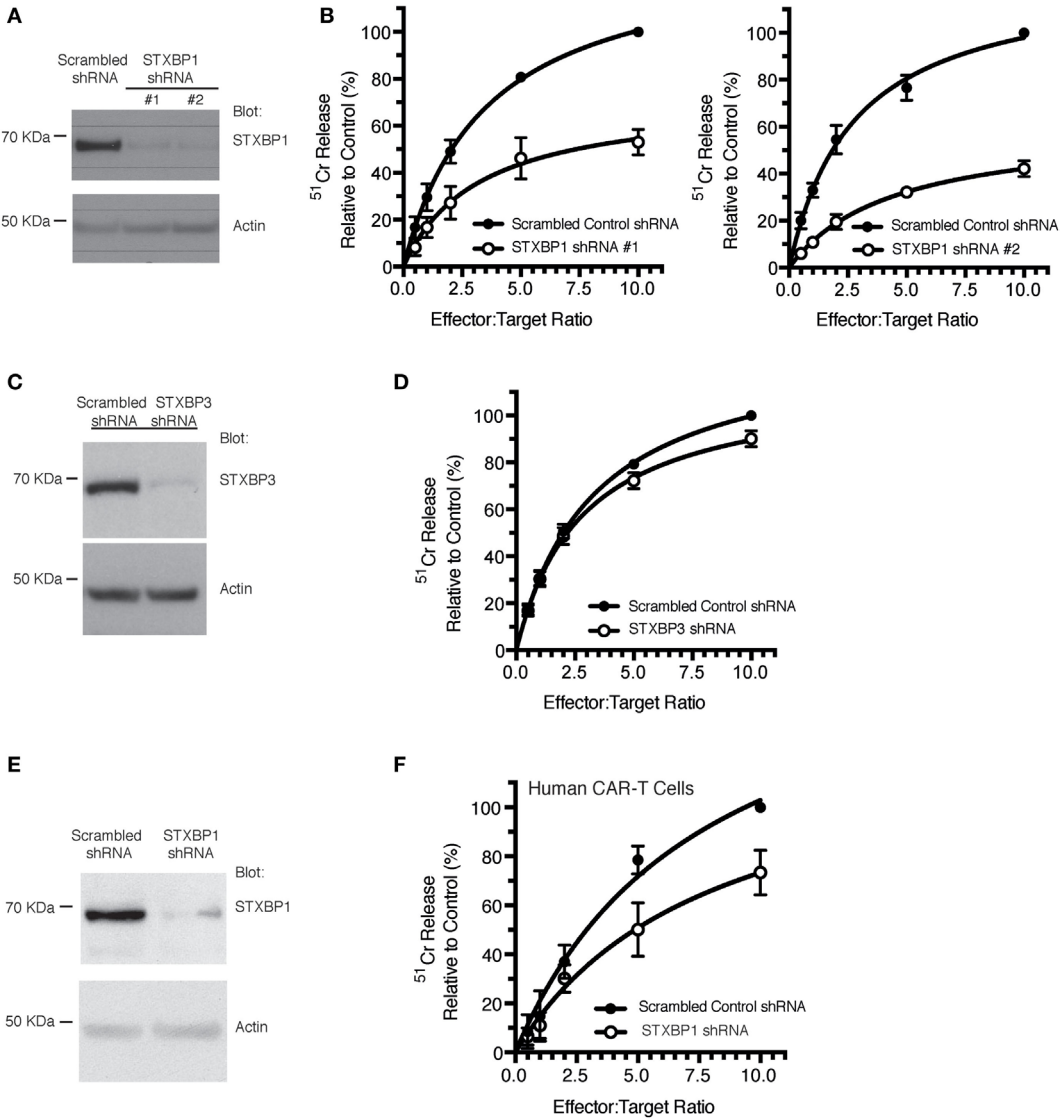
STXBP1 depletion impairs natural killer (NK) cytotoxicity. **(A,B)** KHYG1 cells were transduced with virus expressing a scrambled control shRNA or two different shRNAs targeting STXBP1 (#1, #2). Cells expressing the shRNAs were sorted based on the expression of the BFP fluorescent reporter. **(A)** Whole cell lysates were blotted for STXBP1 and actin (loading control). Molecular weight standards (KDa) are indicated. **(B)** Control and STXBP1 shRNA KHYG1 cell lines were incubated with ^51^Cr-labeled K562 target cells for 4 h at the indicated effector to target cell ratios. Data represent the mean ± SEM (*n* = 3–4 experiments). Data have been normalized to maximal killing observed in the control scrambled shRNA cell line at 10:1 E/T ratio (set at 100%). **(C,D)** KHYG1 cells were transduced with virus expressing a scrambled control shRNA or a shRNA targeting STXBP3. Cells expressing the shRNAs were sorted based on the expression of the GFP fluorescent reporter. **(C)** Whole cell lysates were blotted for STXBP3 and actin (loading control). Molecular weight standards (KDa) are indicated. **(D)** Control and STXBP3 shRNA KHYG1 cell lines were incubated with ^51^Cr-labeled K562 target cells for 4 h at the indicated effector to target cell ratios. Data represent the mean ± SEM (*n* = 7 experiments). Data have been normalized to maximal killing observed in the control scrambled shRNA cell line at 10:1 E/T ratio (set at 100%). **(E,F)** PBMCs were isolated from a healthy donor control, transduced with virus expressing either scrambled shRNA or STXBP1 shRNA and sorted based on the expression of the BFP reporter. BFP+ cells were subsequently transduced with virus expressing the CAR-T. CAR-T− and CAR-T+ cells were sorted based on the expression of the surface expression of the myc epitope. **(E)** Whole cell lysates from the CAR-T− cells were blotted for STXBP1 and actin (loading control). Molecular weight standards (KDa) are indicated. **(F)** CAR-T+ cells were incubated with ^51^Cr-labeled MDA-MB-231 Her2 expressing target cells for 4 h at the indicated effector to target cell ratios. CAR-T cytotoxicity was determined by the release of ^51^Cr from target cells. Data represent the mean ± SEM (*n* = 3 experiments).

To ascertain the role of the STXBP1 protein in CLs function, we used the human NK cell line, KHYG1, which we have previously shown is a suitable model for assessing the NK cell granule death pathway (15). Knockdown of *STXBP1* using two different shRNAs resulted in a >90% reduction in STXBP1 protein levels (Figure 3A), and both knockdown cell lines had significantly decreased NK cell-mediated killing of target cells compared with control cell lines (Figure 3B). We observed no significant loss of function in *STXBP2* knockdown NK cells, which was likely due to the presence of IL-2 (600 U/mL) in the culture media (Figures S1A,B in Supplementary Material). *STXBP3* knockdown also had no effect on NK cell function (Figures 3C,D), which demonstrates STXBP isoform specificity in the CL-mediated killing pathway. To substantiate our findings further, we knocked down *STXBP1* in primary human CD3+CD8+ T cells that were also transduced with anti-HER2 CAR and demonstrated that the STXBP1-deficient T cells had significantly lower cytotoxic function than control T cells (Figures 3E,F).

## Discussion

In the current study, we have made the unexpected observation that congenital deficiency of the STXBP2 protein may also affect the expression of STXBP1. Further analysis identified an unsuspected functional role for STXBP1 in secretory granule-mediated NK and T-cell cytotoxicity.

Granule/vesicle docking and fusion events at the plasma membrane are regulated by at least three Sec1/Munc18-like (SM) isoforms in mammalian cells: STXBP1 (n-Sec1/Munc18-1), STXBP2 (Munc18-2), and STXBP3 (Munc18-3). STXBP1 expression/function was originally thought to be restricted to neuronal tissue (16), however, subsequent work has since reported roles for STXBP1 in other cell types, including pancreatic beta cells (17) and mast cells (18).

There are certain cellular pathways where single SM isoforms have been shown to regulate discrete trafficking events. Loss of STXBP1, for example, results in a complete block in neurotransmission, despite the presence of both STXBP2 and STXBP3 paralogs in neurons (19). By contrast, both STXBP1 and STXBP2 proteins appear to be important in mast cell degranulation, suggesting that their role may be partly redundant or complementary across the hematopoietic cell lineage (18).

In this study, we have serendipitously uncovered a role for STXBP1 as a regulator of NK and cytotoxic T-cell granule exocytosis. One previous study proposed a similar role for this protein based on its biochemical interaction with Syntaxin-11 *in vitro* (14). In the current study, STXBP2 deficiency led to reduced levels of Syntaxin-11 as has been previously reported. However we also observed a concomitant protein reduction in STXBP1 and its partner t-SNARE Syntaxin-1, suggesting a greater protein interdependency between these STXBP/Syntaxin complexes. Our functional analyses revealed that STXBP1 played a role in NK and T-cell cytotoxicity in the presence of IL-2. Since NK and T cells require IL-2 for their growth, we were unable to investigate the role of STXBP1 in the absence of this cytokine. Given the lack of naive NK cell cytotoxicity in the patient described here, we hypothesize that STXBP1 remains “silent” in the absence of IL-2 and only acts in an IL-2 dependent manner to facilitate cytotoxic secretory vesicle docking and target cell killing. Of note, in the current case study, the patient had a significantly reduced level of STXBP1, and this may potentially explain why NK cell function is only partially restored in the presence of IL-2.

STXBP1 plays a non-redundant role in neurotransmission: genetic deletion of STXBP1 results in embryonic lethality in mice (19), and *de novo* mono-allelic mutations in humans invariably result in epileptic encephalopathy (a rare form of Dravet syndrome) (20). It is, therefore, not surprising that STXBP1 mutations have never been associated with primary immunodeficiency. Importantly, in the current study, the loss of STXBP1 protein was greater than 70% (Figure 2C), strongly suggesting that the observed phenomenon is cell-type specific. Interestingly, a link between STXBP1 and STXBP2 protein expression has been previously described *in vitro* (21), where loss of STXBP1 resulted in an upregulation of STXBP2 protein, potentially as a compensatory event. By contrast, at least in the current clinical case, the loss of STXBP2/syntaxin-11 also destabilized the STXBP1/syntaxin-1 protein complex, suggesting that there may be a greater interplay between individual Munc18/Syntaxin complexes that act in concert to orchestrate the same exocytic event.

## Concluding Remarks

In the current study, we have identified STXBP1 as an important player in CL function. Although it did not compensate for the loss of STXBP2 in naïve NK cells, STXBP1 appears to play an important role in activated NK and cytotoxic T cells and may be responsible for as much as 50% of their cytotoxic activity. Future studies using a larger cohort of individuals with *STXBP2* mutations will determine whether the reduction in STXBP1 protein levels in our STXBP2-deficient FHL case is a more general phenomenon, and will help to unravel the complexity of CL secretory machinery.

## Ethics Statement

This study was carried out in accordance with the recommendations of the Peter MacCallum Cancer Centre Human Ethics Committee (approval number 12/73). All subjects gave written informed consent in accordance with the Declaration of Helsinki.

## Author Contributions

JAL, TN, LL, KT, HA, PKD, MHK, JAT and IV conducted experiments and/or interpreted the data, and reviewed and revised the manuscript; AM, NJB, AG diagnosed and treated the patient, interpreted the data; JAL, TN, NJB, AG, JAT, IV co-wrote the manuscript; MSH conducted experiments, reviewed manuscript, interpreted the data, and agreed with all aspects of the work; all authors approved the manuscript and agreed with all aspects of the work.

## Conflict of Interest Statement

The authors declare that the research was conducted in the absence of any commercial or financial relationships that could be construed as a potential conflict of interest. The reviewer GB and handling editor declared their shared affiliation.

## References

[1] Lopez JA Brennan AJ Whisstock JC Voskoboinik I Trapani JA. Protecting a serial killer: pathways for perforin trafficking and self-defence ensure sequential target cell death. Trends Immunol (2012) 33(8):406–12. 10.1016/j.it.2012.04.001 22608996

[2] de Saint Basile G Ménasché G Fischer A. Molecular mechanisms of biogenesis and exocytosis of cytotoxic granules. Nat Rev Immunol (2010) 10:568–79. 10.1038/nri2803 20634814

[3] Cote M Menager MM Burgess A Mahlaoui N Picard C Schaffner C Munc18-2 deficiency causes familial hemophagocytic lymphohistiocytosis type 5 and impairs cytotoxic granule exocytosis in patient NK cells. J Clin Invest (2009) 119(12):3765–73. 10.1172/JCI40732 19884660 PMC2786810

[4] Zur Stadt U Rohr J Seifert W Koch F Grieve S Pagel J Familial hemophagocytic lymphohistiocytosis type 5 (FHL-5) is caused by mutations in Munc18-2 and impaired binding to syntaxin 11. Am J Hum Genet (2009) 85(4):482–92. 10.1016/j.ajhg.2009.09.005 19804848 PMC2756548

[5] Spessott WA Sanmillan ML McCormick ME Kulkarni VV Giraudo CG. SM protein Munc18-2 facilitates transition of Syntaxin 11-mediated lipid mixing to complete fusion for T-lymphocyte cytotoxicity. Proc Natl Acad Sci U S A (2017) 114(11):E2176–85. 10.1073/pnas.1617981114 28265073 PMC5358394

[6] Bryceson YT Rudd E Zheng C Edner J Ma D Wood SM Defective cytotoxic lymphocyte degranulation in syntaxin-11 deficient familial hemo-phagocytic lymphohistiocytosis 4 (FHL4) patients. Blood (2007) 110(6):1906–15. 10.1182/blood-2007-02-074468 17525286 PMC1976360

[7] Jessen B Kogl T Sepulveda FE de Saint Basile G Aichele P Ehl S. Graded defects in cytotoxicity determine severity of hemophagocytic lymphohistiocytosis in humans and mice. Front Immunol (2013) 4:448. 10.3389/fimmu.2013.00448 24379813 PMC3864253

[8] Pagel J Beutel K Lehmberg K Koch F Maul-Pavicic A Rohlfs AK Distinct mutations in STXBP2 are associated with variable clinical presentations in patients with familial hemophagocytic lymphohistiocytosis type 5 (FHL5). Blood (2012) 119(25):6016–24. 10.1182/blood-2011-12-398958 22451424

[9] Newbold A Matthews GM Bots M Cluse LA Clarke CJ Banks KM Molecular and biologic analysis of histone deacetylase inhibitors with diverse specificities. Mol Cancer Ther (2013) 12(12):2709–21. 10.1158/1535-7163.MCT-13-0626 24092806

[10] Matthews GM Mehdipour P Cluse LA Falkenberg KJ Wang E Roth M Functional-genetic dissection of HDAC dependencies in mouse lymp-hoid and myeloid malignancies. Blood (2015) 126(21):2392–403. 10.1182/blood-2015-03-632984 26447190 PMC4653767

[11] Haynes NM Trapani JA Teng MW Jackson JT Cerruti L Jane SM Single-chain antigen recognition receptors that costimulate potent rejection of established experimental tumors. Blood (2002) 100(9):3155–63. 10.1182/blood-2002-04-1041 12384413

[12] House IG House CM Brennan AJ Gilan O Dawson MA Whisstock JC Regulation of perforin activation and pre-synaptic toxicity through C-terminal glycosylation. EMBO Rep (2017) 18(10):1775–85. 10.15252/embr.201744351 28808112 PMC5623865

[13] Sutton VR Waterhouse NJ Baran K Browne K Voskoboinik I Trapani JA Measuring cell death mediated by cytotoxic lymphocytes or their granule effector molecules. Methods (2007) 44:241–9. 10.1016/j.ymeth.2007.11.011 18314055

[14] Hackmann Y Graham SC Ehl S Honing S Lehmberg K Arico M Syntaxin binding mechanism and disease-causing mutations in Munc18-2. Proc Natl Acad Sci U S A (2013) 110(47):E4482–91. 10.1073/pnas.1313474110 24194549 PMC3839780

[15] Chia J Thia K Brennan AJ Little M Williams B Lopez JA Fatal immune dysregulation due to a gain of glycosylation mutation in lymphocyte perforin. Blood (2012) 119(7):1713–6. 10.1182/blood-2011-08-374355 22186995

[16] Hata Y Slaughter CA Sudhof TC. Synaptic vesicle fusion complex contains unc-18 homologue bound to syntaxin. Nature (1993) 366(6453):347–51. 10.1038/366347a0 8247129

[17] Mandic SA Skelin M Johansson JU Rupnik MS Berggren PO Bark C. Munc18-1 and Munc18-2 proteins modulate beta-cell Ca2+ sensitivity and kinetics of insulin exocytosis differently. J Biol Chem (2011) 286(32):28026–40. 10.1074/jbc.M111.235366 21690086 PMC3151048

[18] Bin NR Jung CH Piggott C Sugita S. Crucial role of the hydrophobic pocket region of Munc18 protein in mast cell degranulation. Proc Natl Acad Sci U S A (2013) 110(12):4610–5. 10.1073/pnas.1214887110 23487749 PMC3607013

[19] Verhage M Maia AS Plomp JJ Brussaard AB Heeroma JH Vermeer H Synaptic assembly of the brain in the absence of neurotransmitter secretion. Science (2000) 287(5454):864–9. 10.1126/science.287.5454.864 10657302

[20] Carranza Rojo D Hamiwka L McMahon JM Dibbens LM Arsov T Suls A De novo SCN1A mutations in migrating partial seizures of infancy. Neurology (2011) 77(4):380–3. 10.1212/WNL.0b013e318227046d 21753172 PMC3140798

[21] Arunachalam L Han L Tassew NG He Y Wang L Xie L Munc18-1 is critical for plasma membrane localization of syntaxin1 but not of SNAP-25 in PC12 cells. Mol Biol Cell (2008) 19(2):722–34. 10.1091/mbc.E07-07-0662 18077557 PMC2230596

