# Supplementary material for: Bi-Allelic Mutations in STXBP2 Reveal a Complementary Role for STXBP1 in Cytotoxic Lymphocyte Killing
Source: Front Immunol. 2018 Mar 15;9:529. doi: 10.3389/fimmu.2018.00529 (PMC5862791; doi:10.3389/fimmu.2018.00529)
Supplement: Supplementary file 1 [file data_sheet_1.PDF]

Supplemental Figure 1.

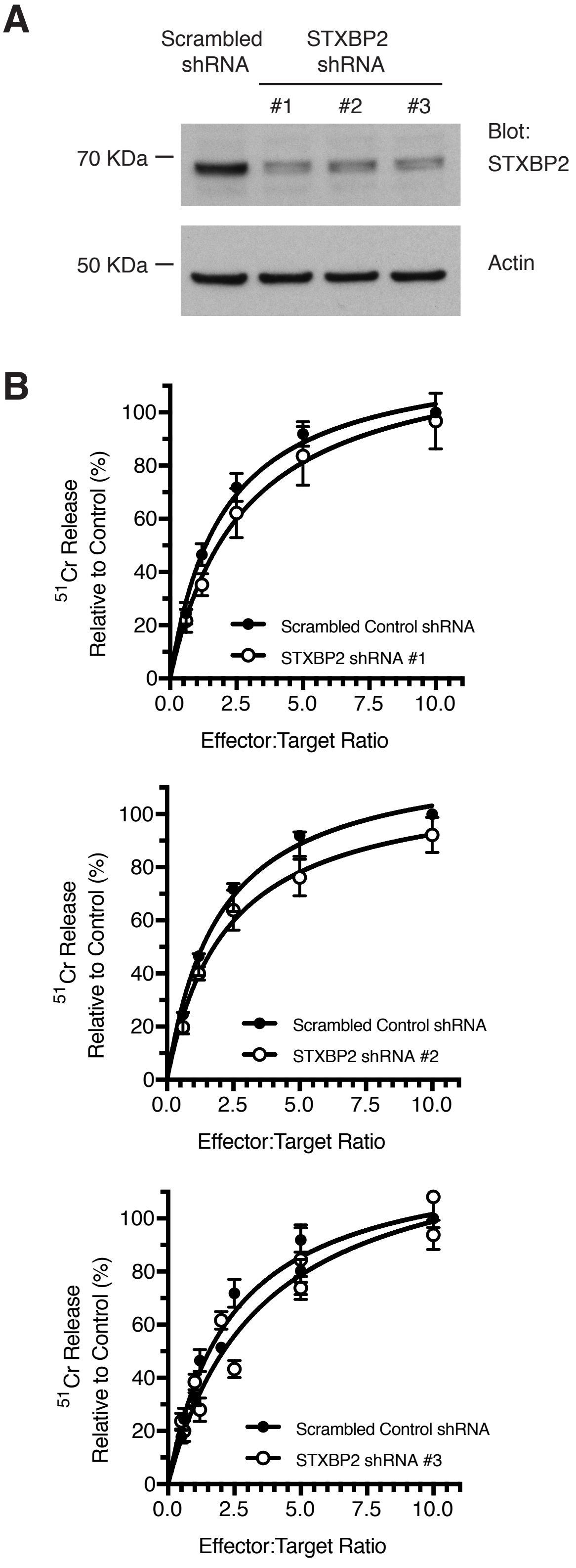

STXBP2 knockdown has no effect on NK cytotoxicity *in vitro*.

KHYG1 cells were transduced with virus expressing a scrambled control shRNA or three different shRNAs targeting STXBP2 (#1, #2, #3). Cells expressing the shRNAs were sorted based on the expression of the mCherry fluorescent reporter. (A) Whole cell lysates were blotted for STXBP2 and actin (loading control). Molecular weight standards (KDa) are indicated. (B) Control and

STXBP2 shRNA KHYG1 cell lines were incubated with ^{51}Cr-labelled K562 target cells for 4 h at the indicated effector to target cell ratios. Data represent the mean ± S.E.M (n = 4-7 experiments). Data have been normalised to maximal killing observed in the control cell line (set at 100%).

Supplemental Figure 2.

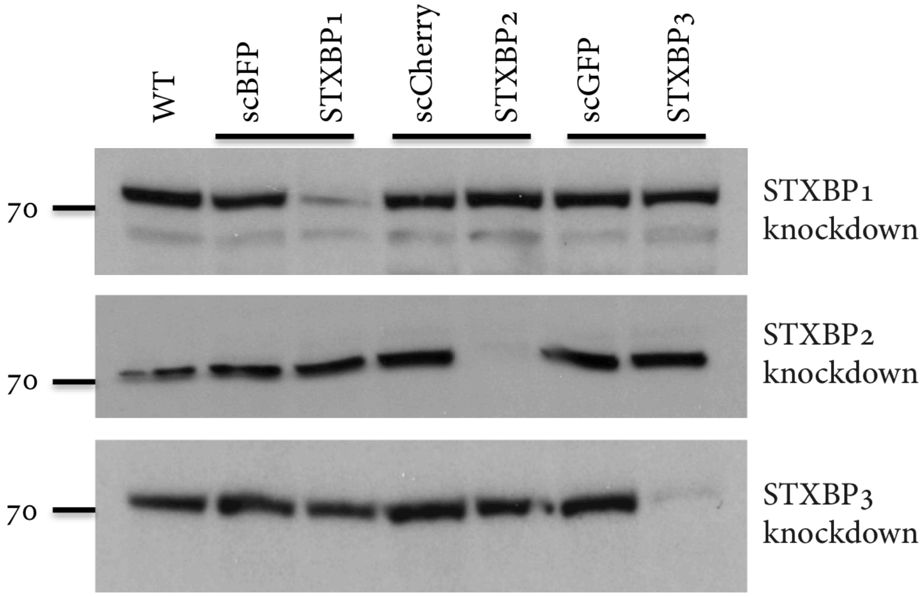

Gene-specific knockdown of STXBP1, STXBP2 and STXBP3 in KHYG1 cells.
STXBP1, STXBP2 or STXBP3 knockdown KHYG1 cells have a normal level of expression of their paralogues that were not targeted by shRNA. Also shown are matching scrambled shRNA controls.
